# Early Postnatal Home Visit Coverage by Health Extension Workers and Associated Factors Among Postpartum Women in Gidan District, Northeast Ethiopia

**DOI:** 10.3389/ijph.2023.1605203

**Published:** 2023-04-03

**Authors:** Desale Bihonegn Asmamaw, Tadele Biresaw Belachew, Abel Endawkie, Wubshet Debebe Negash

**Affiliations:** ^1^ Department of Reproductive Health, Institute of Public Health, College of Medicine and Health Sciences, University of Gondar, Gondar, Ethiopia; ^2^ Department of Health Systems and Policy, Institute of Public Health, College of Medicine and Health Sciences, University of Gondar, Gondar, Ethiopia; ^3^ Department of Epidemiology and Biostatistics, School of Public Health, College of Medicine and Health Science, Wollo University, Dessie, Ethiopia

**Keywords:** postnatal home visits, health extension workers, postpartum women, Gidan district, Ethiopia

## Abstract

**Objectives:** To determine the coverage and associated factors of early postnatal home visits (PNHVs) by health extension workers (HEWs) among postpartum women in Gidan district, Northeast Ethiopia.

**Methods:** A community-based, cross-sectional study was conducted between 30 March and 29 April 2021 in the Gidan district, Northeast Ethiopia. A multistage sampling technique was employed to select 767 postpartum women participants. Interviewer-administered questionnaires were used to collect the data. A binary logistic regression model was fitted to identify factors associated with early PNHVs by HEWs.

**Results:** The coverage of early postnatal home visits was 15.13% [95% confidence interval (CI): 12.75, 17.87]. Women’s education, institutional delivery, time to reach health posts, and participation in pregnant women forums were significantly associated with early PNHVs by HEWs.

**Conclusion:** In the current study, the coverage of early postnatal home visits by HEWs remains low in the study area. The concerned bodies should consider interventions that promote women’s education and institutional delivery, and more efforts should be made to improve community-based participation and links with HEWs.

## Introduction

The majority of maternal and neonatal deaths occur in the first 6 weeks following delivery, especially the first 3 days (1). Worldwide, approximately 4 million neonates and 287,000 maternal deaths occur each year due to maternal and newborn complications within 24 h of birth, and the majority of these deaths occur in developing countries, including Ethiopia (2, 3). Sub-Saharan Africa (SSA) and Southern Asia account for approximately 86% of all maternal and neonatal deaths worldwide (3). The maternal mortality ratio (MMR) and neonatal mortality in Ethiopia are 412 per 1,000 live births and 30 per 1,000 live births, respectively, which remain the highest in the world (4).

Furthermore, scholars revealed that most factors that lead to neonatal deaths can be averted through early postnatal care (5–8). Postnatal care for both mother and baby during the first few weeks after birth is very important, as it can help identify danger signs as well as providing health counseling (9). According to the World Health Organization (WHO), a significant number of women prefer to return home or are discharged within a few hours of delivery, which prevents them from receiving postnatal care, particularly those living in remote areas. To overcome these problems, early postnatal home visits are essential (1, 10).

The United Nations Global Strategy for Women, Children, and Adolescent Health 2016–2030 aims to reduce the national maternal mortality ratio and the neonatal mortality rate to 70 per 100,000 live births and 12 per 1,000 live births, respectively (11). The strategy strives for a world in which mothers and newborns have equal opportunities to survive and thrive (12).

In Ethiopia, health extension programs launched in 2003 aimed to mobilize community members to seek antenatal and neonatal care. Health extension workers (HEWs) were trained to provide basic maternal and child healthcare and to improve the utilization of maternal healthcare such as antenatal care, place of delivery, and postnatal care. HEWs are expected to spend 75% of their time in the community providing essential health services through house-to-house visits (10, 13).

Routine home visits by HEWs during the postnatal period consider the identification, assessment, management, and referral of both mother and infant for care. A mother’s postnatal care services at home include assessing her general condition, checking her vital signs, and monitoring for danger signs (10). For newborns, it includes a general body examination, checking for general danger signs, checking the umbilical cord stump for any bleeding or infection, and assessing breastfeeding (10).

Studies on postnatal care within 48 h revealed low coverage (14–17). The coverage by community health workers (CHWs) within 3 days after delivery in three countries (Bangladesh, Nepal, and Malawi) was 57, 50, and 11%, respectively (15). Studies conducted in the rural Tigray region of Ethiopia and southern Ethiopia revealed that 14.5% and 12.4%, respectively, of mothers and their neonates visited by HEWs received PNHVs within 3 days of birth and during the first month after birth (10, 17).

The literature reports that the following factors affect PNHVs for mothers who received at least one home visit during pregnancy: mothers participating in pregnant women’s forums, members of community health insurance, skilled delivery, the HEW’s cell phone number, and receiving birth notifications from HEWs (5, 9, 18). However, factors influencing early PNHVs by HEWs vary from place to place depending on the culture and socioeconomic status of a given society. Thus, assessing the factors influencing early PNHVs by HEWs in the study area is important for designing public health interventions to improve coverage of early PNHVs by HEWs. Therefore, the aim of this study was to determine the coverage and associated factors of early PNHVs among postpartum women.

## Methods

### Study Design, Period, and Setting

A community-based cross-sectional study design was conducted in the Gidan district, North Wollo zone, Amhara region, Northeast Ethiopia. The district is located 595 km from Addis Ababa, the capital city of Ethiopia, and it has two urban and 21 rural kebeles (the lowest administrative unit). It has an estimated population of 148,058 based on population projection from the 2007 census through to 2020, of which 74,461 and 29,649 are females and reproductive-age women, respectively (19). The district has six health centers and 23 health posts that provide routine health services for the catchment population (19). The study was conducted from 30 March to 29 April 2021.

### Study Population, Sample Size Determination, and Sampling Procedures

All mothers who gave birth within the last year in the Gidan district were the source population. The study population included all mothers who had given birth within the previous year and lived in the district’s selected kebeles.

The required sample size for early postnatal home visits was calculated using the single population proportion formula, with the following statistical assumptions: a 3% margin of error (0.03), a Z-value of 1.96 corresponding to a 95% confidence level, a design effect of 1.5, and a 12.4% coverage of the early postnatal home visits in the rural Sidama Zone of southern Ethiopia as determined in a previous study (17). Accordingly, the sample size was computed as follows:

$$n=\frac{{\left(Z\alpha 1/2\right)}^{2}P\left(1-P\right)}{d^{2}}$$

$$n=\frac{{\left(1.96\right)}^{2}\left(0.124\right)\left(1-0.124\right)}{{\left(0.03\right)}^{2}}=464$$

n=464

After adding a 10% non-response rate and multiplying the result by the design effect of 1.5, the total sample size of this study was 767 mothers.

A multistage sampling method with stratification of the district into rural and urban areas was used based on residence. Gidan district has 21 rural and two urban kebeles, and from those, 30% of the total kebeles (six kebeles from the rural and one kebele from the urban) were selected using a simple random sampling technique. The list of mothers who gave birth in the last year was identified by the health center or health extension workers. The sample size was proportionally allocated to each selected kebele considering the number of women. Mothers in the sampled kebeles were selected by using a simple random sampling technique (Open Epi Random Program version 3). If the respondents were not available at home during the time of data collection, interviewers revisited the households three times, and when the interviewers failed to find the eligible respondent after three visits, the next household was included.

### Study Variables

The outcome variable was an early postnatal home visit by a HEW. The coverage of early postnatal home visits was defined as the percentage of mothers and/or newborns that were visited at home within 3 days after delivery (10, 17). HEWs are a cadre of government workers who received 1 year of training and are paid a government salary to deliver the health program at the community level in rural areas (20).

The independent variables were socio-demographic (educational status of women and their husbands, marital status, residence, and time taken to reach the health post), obstetrics-related characteristics (parity, ANC visit by the HEWs, participation in pregnant women forums, birth notification, and place of delivery), and other characteristics such as membership of the Women’s Development Army (WDA) and HEWs’ cell phone availability.

### Data Quality Control

Enumerators and supervisors were trained for 2 days, focusing on how to ask the question on and fill out the questionnaires, the selection criteria for women, and how to approach the participants. Before starting the actual data collection, the data collectors practiced in the field, and the questionnaires were pretested on 40 study participants (5%) in the Gubalafto district. Findings and experiences from the pretest were utilized to modify the data collection tool. The data collectors and the principal investigator assessed the clarity and completeness of the completed questionnaires. The whole data collection process was closely supervised by the principal investigator (PI).

### Data Collection Tools and Procedures

Structured interviewer-administered questionnaires, developed by reviewing different related studies on different regions of Ethiopia, were used for data collection (17, 18, 21). Firstly, the questionnaires were developed in English. Then, they were translated into Amharic (the local language) and retranslated back into English to check for consistency. The questionnaires were separated into different sections such as socio-demographic characteristics, obstetric-related characteristics, and other characteristics such as membership of WDA and having the HEW’s cell phone number. Eight BSc-qualified midwifery/nursing data collectors and two BSc-qualified midwife supervisors with experience in research and fieldwork coordination participated in the data collection process.

### Data Processing and Analysis

The collected data were checked for completeness and consistency by supervisors and principal investigators. The data were entered into Epi-Data Statistical Software version 4.6. Then, the data were exported to Stata 14 Statistical Software for cleaning, coding, and analysis. Descriptive statistics were described using frequencies, percentages, means, and standard deviations, which were further presented using tables, figures, and text. Normality tests such as kurtosis and skewness were employed to determine the normal distribution of the variables and to identify which summary measures were appropriate to use.

Binary logistic regression analysis was carried out to identify factors associated with early PNHVs by HEWs. Variables with a *p*-value ≤0.25 from the bivariable analysis were entered into a multivariable logistic regression model to control the possible effects of confounders. Before performing multivariable logistic regression, we computed Hosmer and Lemeshow’s goodness of fit, and the model was adequate, with a *p*-value of 0.94. Moreover, multicollinearity was tested using the variance inflation factor (VIF), and a VIF of less than five was obtained for each independent variable, with a mean VIF of 1.55, indicating that there was no significant multicollinearity between independent variables. The odds ratio with 95% confidence intervals was computed to check if there was an association between early PNHVs by HEWs and associated factors. A *p*-value of 0.05 was considered to show a statistical association.

## Results

### Socio-Demographic Characteristics of the Participants

Approximately 760 mothers participated, indicating a response rate of 99.93%. The mean age of the study participants was 27 (SD ± 5.3) years, and 305 (40.13%) fell within the age category of 25–29 years. Three-quarters (75.39%) of the respondents were rural dwellers, and almost all (99.61%) participants were orthodox Christian (Table 1).

**TABLE 1:** Socio-demographic characteristics of the respondents in Gidan district, Northeast Ethiopia (2021, N = 760).

Variable	Frequency	Percentage
Maternal age		
18–24	241	31.71
25–29	305	40.13
30–39	198	26.05
40–49	16	2.11
Residence		
Rural	573	75.39
Urban	187	24.61
Religion		
Orthodox	757	99.61
Muslim	3	0.39
Education of the mothers		
No formal education	366	48.16
Primary education	289	38.03
Secondary education and above	105	13.82
Occupation of the respondents		
Housewife	645	84.87
Government employed	86	11.32
Self employed	21	2.76
Other^a^	8	1.05
Marital status		
Married	671	88.29
Unmarried	89	11.71
Sex of the newborn		
Male	322	42.37
Female	438	57.63
Educational status of the husbands		
No formal education	368	54.84
Primary education	196	29.21
Secondary education and above	107	15.95
Occupation of the husbands		
Farmer	546	81.37
Government employed	56	8.30
Self employed	53	7.90
Other^b^	16	2.43

^a^ Student.

^b^ Daily labor/soldier.

### Obstetrics and Other Related Factors

Among the study participants, 612 (80.53%) and 105 (13.82%) of the mothers were multiparous and had ANC visits by the HEWs, respectively. One hundred and fifty-six (20.53%) of the participants were members of the Women’s Development Army (WDA). Approximately 541 (71.18%) of the mothers delivered their babies at health institutions (Table 2).

**TABLE 2:** Obstetrics and other related factors of the respondents in Gidan district, Northeast Ethiopia (2021, N = 760).

Variable	Frequency (n)	Percentage (%)
Parity		
Primipara	148	19.47
Multi para	612	80.53
Membership of women’s development army		
Yes	156	20.53
No	604	79.47
Participation in pregnant women forums		
Yes	376	49.47
No	384	50.53
ANC visit		
Yes	105	13.82
No	655	86.18
Place of delivery		
Home	219	28.82
Health institutions	541	71.18
HEW cell phones		
Yes	148	19.47
No	612	80.53
Distance to health facilities		
<30 min	366	48.16
30 min–1 h	249	32.76
>1 h	145	19.08

### Early Postnatal Home Visit Coverage

**FIGURE 1:**
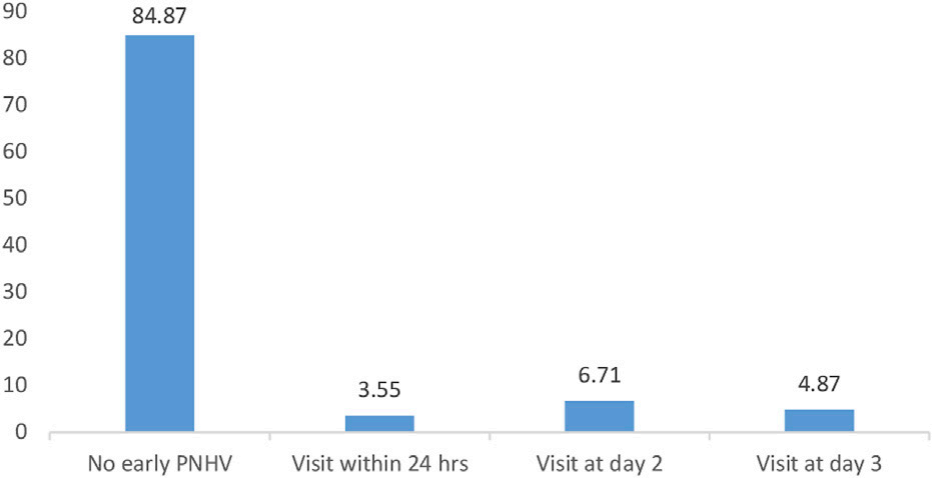
Proportion of mothers who received a PNHV within 3 days after birth. Northeast, Ethiopia (2021, *N* = 760).

The coverage of early PNHVs in the Gidan district was 15.13% (95% CI = 12.75, 17.87). Of these, 3.55% of the mothers and their neonates received a PNHV within 24 h (Figure 1).

### Factors Associated With Early Postnatal Home Visits

Based on the multivariable logistic regression analysis, the mother’s education, place of delivery, time taken to travel the health post, and participation in pregnant women forums were statistically significant factors for early PNHVs. Accordingly, an early PNHV was 1.84 and 3.77 times higher for individuals with primary and secondary education or above (AOR = 1.84, 95% CI: 1.06, 3.19) and (AOR = 3.77, 95% CI: 1.81, 7.88), respectively, compared to individuals with no formal education.

Similarly, the odds of women having an early PNHV were 5.34 (AOR = 5.34, 95% CI: 2.39, 12.08) times higher among mothers who gave birth at health institutions compared to women who gave birth at home. Mothers with <30 min taken to reach the health post were 2.58 times (AOR = 2.58, 95% CI: 1.15, 5.82) more likely to have an early PNHV compared to their counterparts. The likelihood of an early PNHV was 2.19 times higher (AOR = 2.19, 95% CI: 1.37, 3.52) among respondents who participated in pregnant women forums as compared to their counterparts (Table 3).

**TABLE 3:** Multi-variable regression for factors associated with home delivery in Northeast, Ethiopia (2021, N = 760).

Variable	PPHV		COR (95%CI)	AOR (95%CI)
	Yes	No		
Residence				
Rural	86	487	1	1
Urban	29	158	1.04 (0.66, 1.64)	0.81 (0.46, 1.43)
Education of the mother				
No formal education	36	330	1	1
Primary education	50	239	1.92 (1.21, 3.04)	1.84 (1.06, 3.19)
Secondary education and above	29	76	3.51 (2.02, 6.06)	3.77 (1.81, 7.88)
Marital status				
Married	105	566	1.47 (0.74, 2.92)	1.86 (0.21, 16.05)
Unmarried	10	79	1	1
Husband education				
No formal education	54	321	1	1
Primary education	33	169	1.16 (0.72, 1.86)	0.91 (0.53, 1.56)
Secondary education and above	17	93	1.09 (0.60, 1.96)	0.56 (0.25, 1.23)
Parity				
Primipara	23	125	1	1
Multipara	92	520	0.96 (0.59, 1.58)	1.36 (0.75, 2.43)
Place of delivery				
Home	211	8	1	1
Health institutions	434	107	6.5 (3.11, 13.59)	5.34 (2.39, 12.08)
Distance to the health post				
<30 min	75	291	3.89 (1.89, 8.01)	2.58 (1.15, 5.82)
30 min–1 h	31	218	2.15 (0.99, 4.65)	1.53 (0.65, 3.61)
>1 h	9	136	1	1
Membership of WDA				
Yes	27	129	1.23 (0.77, 1.97)	0.93 (0.54, 1.59)
No	88	516	1	1
Participation in pregnant women forums				
Yes	79	297	2.57 (1.68, 3.93)	2.19 (1.37, 3.52)
No	36	348	1	1
HEWs’ cellphones available				
Yes	16	132	1.59 (0.91, 2.79)	1.43 (0.77, 2.64)
No	99	513	1	1
ANC visits by HEWs				
Yes	13	92	0.77 (0.42, 1.43)	1.04 (0.51, 2.11)
No	102	553	1	1

Abbreviations: COR, crude odds ratio; AOR, adjusted odds ratio; WDA, women’s development army; HEW, health extension worker.

## Discussion

Early postnatal home visits by HEWs reduce maternal and newborn complications, especially at higher coverage (16, 22). However, our findings revealed that only 15.13% of mothers and neonates received early PNHVs by HEWs. The findings of this study are consistent with studies conducted in the rural Tigray region of northern Ethiopia (10). However, the result is higher than studies conducted in Ethiopia (17, 23) and Malawi (24). On the other hand, the current finding is very low compared to other studies performed in Mali (25) and Ethiopia (26). This might be due to the involvement of the health extension workers in a variety of activities. The health extension worker program was initially intended to provide subsequent preventive care and curative activities, such as treating diarrhea, sepsis, pneumonia, malaria, and other illnesses; however, more responsibilities were added to the role. As a result, they are much busier than before (27). Moreover, a number of HEWs do not live in their duty station area but instead come from nearby towns; therefore, they do not work full-time (18, 27). This indicates that a high number of infants and mothers are not receiving effective treatment or health counseling for severe infections or serious illnesses, which could potentially be managed at the community level. This raises maternal and neonatal morbidity and mortality (10, 16).

According to the bivariable and multivariable logistic regression analyses, women’s education, place of delivery, participation in pregnant women forums, and time taken to travel to the health post were found to be significantly associated with early PNHVs by HEWs. In this study, women with a formal education were more likely to have an early PNHV by HEWs as compared to women with no formal education. The possible reason for this might be that women with a formal education are more likely to be exposed to the advantages of an early PNHV by HEWs through the media, which increases the number of early PNHVs by HEWs (28, 29). Previous studies have also shown that mothers with a formal education utilize maternal and newborn healthcare services more frequently (17, 30, 31).

Women who delivered at health institutions were 5.34 times more likely to use early PNHVs than those who delivered at home. This finding is in line with a study conducted in the rural Sidama Zone of southern Ethiopia (17). This might be due to the fact that women who delivered at health institutions had greater opportunities to obtain health education on the benefits of an early PNHV at the time of delivery (32). Women who gave birth in health facilities were usually educated and lived close to the health facility, thereby improving the coverage of early PNHVs by HEWs (21, 32).

In this study, women who participated in pregnant women forums were 2.19 times more likely to have an early PNHV by HEWs as compared to women who did not participate in the forums. This finding is incongruent with a study conducted in the rural Tigray region of Ethiopia (10). The possible reason for this might be that women who participated in the pregnant women forums were more likely to discuss their health issues with each other as well as with other healthcare providers such as HEWs. Moreover, scholars have found that, for women who live in an area with advanced pregnant women forum networks, the utilization of maternal healthcare services, including early PNHVs, has improved (33).

The findings of this study revealed that the time to reach the health post was another factor that affected early postnatal home visits by HEWs. Women who took <30 min to reach the health post were 2.58 times more likely to have a PNHV by HEWs than women who took more over hour to reach the health post. The reason behind this might be the unavailability of transportation and inconvenient geographical and seasonal conditions that restricted the HEWs from visiting the women following childbirth (18). Furthermore, the time required to travel to health institutions and lack of transportation are significant deterrents that prevent women from seeking to utilize maternal healthcare services (34).

### Strength and Limitations of the Study

The main strength of the current study is that it is a community-based study and might reflect the actual experiences of the women during the study period. On the other hand, this study has limitations. Even though all possible strategies were applied, such as using women data collectors, providing training for data collectors, employing pretests, using standardized tools, and securing privacy, there might be recall bias due to the data collected from women about their experiences 1 year ago. A cause-and-effect relationship cannot be established due to the cross-sectional nature of the study. Similarly, employing the HEWs’ or health centers’ registration books as a sampling framework may provide a biased estimate because of missing new-delivery mothers.

### Conclusion

In the current study, the coverage of early PNHV by HEWs remains low in the study area. Women’s education, institutional delivery, participation in pregnant women forums, and time to reach the health post were significantly associated with early PNHVs by HEWs. The concerned bodies should consider interventions that promote women’s education and institutional delivery, and more efforts should be made to improve community-based participation and links with HEWs. The concerned bodies should also strengthen the HEWs’ supportive supervision. Future researchers interested in this area should also consider qualitative research, such as why the HEWs that are currently being implemented do not achieve the expected results.

## Abbreviations

ANC, antenatal care; AOR, adjusted odd ratio; CI, confidence interval; COR, crude odd ratio; HEW, health extension workers; OR, odds ratio; PNC, postnatal care; PNHV, postnatal home visit; WDA, women’s development army; WHO, world health organization.
